# Enhancing Gentamicin Antibacterial Activity by Co-Encapsulation with Thymoquinone in Liposomal Formulation

**DOI:** 10.3390/pharmaceutics16101330

**Published:** 2024-10-15

**Authors:** Raghad R. Alzahrani, Manal M. Alkhulaifi, Majed Al Jeraisy, Abdulkareem M. Albekairy, Rizwan Ali, Bahauddeen M. Alrfaei, Salleh N. Ehaideb, Ahmed I. Al-Asmari, Sultan Al Qahtani, Abdulaziz Halwani, Alaa Eldeen B. Yassin, Majed A. Halwani

**Affiliations:** 1 Department of Botany and Microbiology, College of Science, King Saud University, Riyadh 11451, Saudi Arabia; raghad.r.alzahrani@gmail.com (R.R.A.); manalk@ksu.edu.sa (M.M.A.); 2 Nanomedicine Department, King Abdullah International Medical Research Center, King Saud bin Abdulaziz University for Health Sciences, Riyadh 11481, Saudi Arabia; 3 King Abdullah International Medical Research Center, King Saud bin Abdulaziz University for Health Sciences, Riyadh 11481, Saudi Arabia; jeraisym@ngha.med.sa (M.A.J.); halwani560@ksau-hs.edu.sa (A.H.); 4 Department of Pharmacy Practice, College of Pharmacy, King Abdullah International Medical Research Center, King Saud bin Abdulaziz University for Health Sciences, Riyadh 11481, Saudi Arabia; bekairya@ksau-hs.edu.sa; 5 Pharmaceutical Care Department, King Abdulaziz Medical City, National Guard Health Affairs, Riyadh 11481, Saudi Arabia; 6 Medical Research Core Facility and Platforms, King Abdullah International Medical Research Center, King Saud bin Abdulaziz University for Health Sciences, Riyadh 11481, Saudi Arabia; aliri@kaimrc.edu.sa; 7 Stem Cells and Regenerative Medicine, King Abdullah International Medical Research Center, King Saud bin Abdulaziz University for Health Sciences, National Guard Health Affairs, Riyadh 11481, Saudi Arabia; alrfaeiba@ngha.med.sa; 8 Experimental Medicine Department, King Abdullah International Medical Research Center, King Saud bin Abdulaziz University for Health Sciences, Ministry of National Guard—Health Affairs, Riyadh 11481, Saudi Arabia; ehaidebs@mngha.med.sa; 9 Department of Pathology and Laboratory Medicine, King Faisal Specialist Hospital and Research Centre, Riyadh 11211, Saudi Arabia; 10 Department of Basic Medical Sciences, College of Medicine, King Abdullah International Medical Research Center, King Saud bin Abdulaziz University for Health Sciences, Riyadh 11481, Saudi Arabia; qahtanis@ksau-hs.edu.sa; 11 College of Dentistry, King Saud bin Abdul Aziz University for Health Sciences, Riyadh 11481, Saudi Arabia; 12 College of Pharmacy, King Abdullah International Medical Research Center, King Saud bin Abdulaziz University for Health Sciences, Riyadh 11481, Saudi Arabia

**Keywords:** gentamicin, thymoquinone, liposomes, biofilm, liposomal–bacterial interaction, TEM

## Abstract

Background and Purpose. Gentamicin (GEN) is a broad-spectrum antibiotic that cannot be prescribed freely because of its toxicity. Thymoquinone (THQ), a phytochemical, has antibacterial, antioxidant, and toxicity-reducing properties. However, its hydrophobicity and light sensitivity make it challenging to utilize. This incited the idea of co-encapsulating GEN and THQ in liposomes (Lipo-GEN-THQ). Method. Lipo-GEN-THQ were characterized using the zeta-potential, dynamic light scattering, Fourier transform infrared spectroscopy, and transmission electron microscope (TEM). The liposomes’ stability was evaluated under different storage and biological conditions. Lipo-GEN-THQ’s efficacy was investigated by the minimum inhibitory/bactericidal concentrations (MICs-MBCs), time–kill curves, and antibiofilm and antiadhesion assays. Bacterial interactions with the empty and GEN-THQ-loaded liposomes were evaluated using TEM. Results. The Lipo-GEN-THQ were spherical, monodispersed, and negatively charged. The Lipo-GEN-THQ were relatively stable and released GEN sustainably over 24 h. The liposomes exhibited significantly higher antibacterial activity than free GEN, as evidenced by the four-fold lower MIC and biofilm eradication in resistant *E. coli* strain (EC-219). TEM images display how the empty liposomes fused closely to the tested bacteria and how the loaded liposomes caused ultrastructure damage and intracellular component release. An antiadhesion assay showed that the Lipo-GEN-THQ and free GEN (0.125 mg/L) similarly inhibited *Escherichia coli* (EC-157) adhesion to the A549 cells (68% vs. 64%). Conclusions. The Lipo-THQ-GEN enhanced GEN by combining it with THQ within the liposomes, reducing the effective dose. The reduction in the GEN dose after adding THQ may indirectly reduce the toxicity and aid in developing an enhanced and safer form of GEN.

## 1. Introduction

Bacterial resistance to antibiotics, or as scientists call it, “The Silent Pandemic”, is setting hurdles in treating bacterial infections and is not “silent” anymore. Bacterial resistance increases fatality rates, extends hospital stays, and elevates financial costs [1]. In 2019, multidrug-resistant infections caused 4.95 million deaths in 204 countries [2].

GEN belongs to aminoglycosides (AGs)—broad-spectrum antibiotics and the only protein synthesis inhibitor with a bactericidal effect. Their action is caused by their irreversible attachment to the 30S ribosomal subunit, leading to faulty amino acid synthesis and bacterial cell death [3,4]. GEN was limited due to permeability difficulty, resistance, and toxicity (temporary nephrotoxicity or permanent ototoxicity) [4,5]. Still, comparing AGs to other antibiotics (e.g., fluoroquinolone and β-lactam), AGs do not show an alarming increase in resistance [6].

Liposomes are spherical vesicles with an aqueous core and lipid bilayer membrane, which are biocompatible and advantageous in delivering drugs to targeted sites [7,8,9]. Liposomes are known to reduce drug toxicity [10,11]. In the case of liposomal amphotericin B, the reduction in nephrotoxicity was related to targeted delivery (liposome-receptor specificity) and their small size, allowing them to surpass glomerular filtration by the kidney [10]. Another example is the liposomal doxorubicin, a member of the anthracycline antibiotics family used to treat cancer. Compared to its free form (doxorubicin), it maintained anticancer efficiency while significantly reducing associated cardiotoxicity and improving doxorubicin pharmacokinetics [11,12]. Liposomes are also known to reduce antibiotics’ effective minimum inhibitory concentrations compared to their free form [13,14]. An approach to developing liposomal antibiotics is co-encapsulation with other drugs with a toxicity reduction ability, such as thymoquinone [15]. Thymoquinone (THQ), a phytochemical in *Nigella sativa* seeds, has antibacterial, antibiofilm, anti-inflammatory, and antioxidant properties and can reduce drug-associated toxicity [16,17]. Utilizing THQ is a known challenge due to its hydrophobicity and poor solubility; thus, liposomes serve as a favorable carrier to accommodate THQ’s physicochemical properties [15].

Although the emergence of resistance is inescapable, once novel agents are finally used, the time for resistance to develop is unknown—as bacterial evolution is unpredictable [18]. The rationale behind formulating the liposomal gentamicin–thymoquinone (Lipo-GEN-THQ) is to develop a nano-system carrying THQ as a “supporter” compound to improve GEN and THQ characteristics and effectively impact bacteria with a lower concentration, which may aid in decreasing GEN’s toxicity. Therefore, the Lipo-GEN-THQ formulation aimed to develop an enhanced and safer treatment for bacterial infections.

## 2. Materials and Methods

### 2.1. Materials and Reagents

Materials were sourced through local vendors in Riyadh. Thymoquinone (2-isopropyl-5-methyl-1,4-benzoquinone) was obtained from SynQuest Laboratories, Inc. (Alachua, FL, USA), and gentamicin sulfate (GENTAM^®^) from SPIMACO Addwaeih (Riyadh, Saudi Arabia). Cholesterol, DPPC (1,2-dipalmitoyl-sn-glycero-3-phosphocholine), and DSPC (1,2-distearoyl-sn-glycero-3-phosphocholine) from UFC Biotechnology (Amherst, NY, USA) and DMPG (1,2-dimyristoyl-sn-glycero-3-PG; sodium salt) from Cayman Chemical Company (Ann Arbor, MI, USA). Organic solvents were obtained from Fisher Scientific (Waltham, MA, USA), PBS Buffer from Beckman Coulter, Inc. (Chaska, MN, USA), and Triton-X from Loba Chemie™ (Mumbai, India).

### 2.2. Liposomal Formulation

#### Liposomal Gentamicin–Thymoquinone (Lipo-GEN-THQ) Preparation

The Lipo-GEN-THQ were prepared following a modified method [19]. First, we calculated the phospholipid composition using the molar ratio. The phospholipids of DMPG:DPPC:DSPC:Cholesterol (2:2:2:1) were dissolved in methanol and chloroform. The three lipids had varying acyl chain lengths to ensure liposomal stability and fluidity. The thin lipid film was formed using a rotary evaporator (BUCHI, Flawil, Switzerland) at 35 °C and 150 mbar. Then, the flasks were left at room temperature overnight to ensure solvent evaporation before rehydrating the lipid film. Next, the lipid film was rehydrated with 2 mL of GEN: THQ solution dissolved in PBS pH 7.2 (0.5 mg/mL GEN and 1.5 mg/mL THQ). The flasks were vortexed and incubated in a water bath (35 ± 5 °C). Then, liposomes were sonicated (five seconds of on/off pulses for three minutes, at 60% altitude—thrice) using a Branson 550 Ultrasonicator, Brookfield, WI, USA. For preservation, the liposomes were lyophilized (Labconco, Kansas City, MO, USA) at a −20°C shelf temperature and a 0.7 mbar for 48 to 72 h and stored at 4 °C. On the day of each experiment, the Lipo-GEN-THQ were gradually rehydrated (10% at a time) with PBS until the final volume was reached and then centrifuged (10,000 rpm) thrice to remove the unencapsulated GEN. Lastly, the Lipo-GEN-THQ were injected into the Microfluidics (LV1 Microfluidizer^®^ Processor, Westwood, MA, USA) with Z-type (G10Z) champers and a processed pump pressure of 1200 psi (equivalent to 8 MPa).

### 2.3. Liposomal Gentamicin–Thymoquinone (Lipo-GEN-THQ) Characterization

#### 2.3.1. Gentamicin Concentration, Encapsulation Efficacy (%EE), and Drug-Loading Capacity (%LC) 

The GEN concentration was calculated by assessing the GEN inside the liposomes—given that the THQ was added per the molar ratio and that its lipophilic nature allows it to merge within the liposome’s outer wall. A standard calibration curve (R^2^ = 0.9895) of different concentrations of GEN (700–1.37 mg/L) was tested against *Staphylococcus aureus* ATCC 29213. The GEN concentration inside the liposomes was assessed using the agar well-diffusion assay [20]. Equal volumes (100 µL) of the liposomes and 0.2% triton X-100 were incubated at 37 °C (30 min), sonicated (15 min), and then centrifuged (10,000 rpm for 30 min at 4 °C) to collect the released GEN content. Twenty-five microliters of the supernatant were loaded into 6 mm wells in the Muller–Hinton (MH) agar and incubated overnight at 37 °C before calculating the diameter of the inhibition zone (DIZ). The obtained equation was y = 2.8056 ln(x) + 4.4312, where x is the unknown GEN concentration and y is the DIZ. The liposomes’ encapsulation efficacy and drug-loading capacity were calculated as follows [21]:

Encapsulation Efficacy (%) = (Encapsulated GEN concentration _(mg/L)_/Initial GEN concentration _(mg/L)_) × 100

Drug-Loading Capacity (%) = (Encapsulated GEN concentration _(mg/L)_/Lipids concentration _(mg/L)_) × 100

#### 2.3.2. Dynamic Size, Polydispersity Index, and Z-Potential

The Lipo-GEN-THQ and the empty liposomes (loaded with PBS only) were analyzed for their dynamic size, polydispersity index, and Z-potential using the NanoBrook ZetaPALS Particle Size Analyzer, Brookhaven Instruments Corporation, Nashua, NH, USA. The size stability was monitored at room temperature for a week at 0, 24, 48, 90, 114, and 160 h. The samples were left at room temperature and mixed by pipetting before measuring their dynamic size and polydispersity index at the assigned time points. After dilution, the Z-potential was evaluated using an AQ-1190 electrode cell (Lipo-GEN-THQ at 1:1 and empty liposomes at 1:4 in PBS).

#### 2.3.3. Fourier Transform Infrared Spectroscopy (FTIR) Analysis

The FTIR analysis was conducted using a PerkinElmer Spectrum Two Universal ATR, Shelton, CT, USA. The FTIR spectra analyzed the functional groups in the GEN, THQ, Lipo-GEN-THQ, and empty liposomes. Lyophilized samples (GEN, Lipo-GEN-THQ formulation, and empty liposomes) and THQ crystals were placed on the sample compartment and scanned in the range of 400–4000 cm^−1^ with a 4 cm^−1^ spectral resolution.

#### 2.3.4. Biological Stability

The stability of the Lipo-GEN-THQ formulation in different biological media was evaluated by assessing the released gentamicin from the liposomes at different time points over 24 h. Equal volumes of Lipo-GEN-THQ and PBS, plasma, sputum, and bronchoalveolar lavage fluid (BALF) were added to microfuge tubes and incubated at assigned temperatures. The supernatant was collected by centrifugation at 0, 1, 3, 6, 8, 12, and 24 h, which was then loaded into MH agar following the method used above to calculate the GEN concentration. The gentamicin retention percentage was assessed indirectly using the following equation [22]:

GEN Retention (%) = [(initial GEN concentration _(mg/L)_ − released GEN concentration _(mg/L)_)/initial GEN concentration _(mg/L)_] × 100

### 2.4. Transmission Electron Microscopy

#### 2.4.1. Lipo-GEN-THQ Liposome Morphology

The liposomes were visualized using a JEOL JEM-1400, Peabody, MA, USA, at an accelerating voltage of 100–120 kV. A droplet was loaded into a Formvar-coated copper grid, and after five minutes, the excess moisture was absorbed using filter paper. The grid was air-dried for at least three hours before imaging.

#### 2.4.2. Empty Liposome–Bacterial Membrane Fusion

Empty liposome–bacterial membrane fusion was investigated using Mugabe et al.’s [23] method. Cultures (12–18 h) of *Escherichia coli* ATCC 25922 and *E. coli* (EC-157) were suspended in LB broth (OD_625 nm_ adjusted to 0.6) and incubated for an hour in a shaker incubator at 37 °C. A droplet was loaded in a copper grid and allowed to adhere for five minutes before removing the redundant moisture and examining the grid using TEM after air-drying.

#### 2.4.3. Loaded Liposome–Bacterial Interaction

TEM was used to evaluate the morphological changes in a clinical strain of *E. coli* (EC-157) exposed to the MICs of the formula Lipo-GEN-THQ (1 mg/L) and free GEN (2 mg/L) and compared to untreated cells. The loaded liposome–bacterial interactions were studied using a modified version of Nguyen et al.’s [24] protocol to prepare the bacterial inoculum. Bacterial cells were resuspended and washed twice with PBS (pH 7.0). Next, we followed the procedure of Kim and Rhee (2013) to embed the samples for TEM imaging [25]. Cells were fixed at 4 °C with 2.5% glutaraldehyde and 4% formaldehyde and post-fixed with 1% osmium tetroxide (O_s_O_4_; Polysciences, Inc., Warrington, PA, USA). The cells were dehydrated in ethanol gradients and embedded in epoxy (Sigma-Aldrich, St. Louis, MA, USA). Ultrathin sections were obtained using an Ultramicrotome (RMC PowerTomes, Urbana, IL, USA) and stained with uranyl acetate. Two independent experiments were performed.

### 2.5. Biological Activity

#### 2.5.1. Tested Bacteria and Growth Conditions

All bacterial isolates were obtained anonymously after routine procedures from the Clinical Microbiology Laboratory at the Department of Pathology and Laboratory Medicine, King Abdelaziz Medical City, Riyadh. The bacteria were cultured from a glycerol stock on Luria–Bertani (LB) agar and incubated at 37 °C for 18–20 h.

#### 2.5.2. Free Drugs Checkerboard Assay

The checkerboard assay evaluated the effect of combining thymoquinone (Drug A) and gentamicin (Drug B), mainly to exclude antagonism. The drug interactions can be synergistic, antagonistic, or indifferent (i.e., noninteracting) [26]. The assay was conducted in 96-well microplates following Bellio’s method with minor modifications to the final assessment technique [27]. Different concentrations were tested for THQ and GEN, and the results were compared to the MICs of the free drugs on the same plate. Then, the plate was inoculated with an adjusted bacterial suspension to attain the recommended final concentration (5 × 10^5^ CFU/mL) in each well. After incubation, the plates were assessed visually using black and white lined paper, and the fractional inhibitory concentration index (FICI) was calculated as follows:

FICI = (drug A_(combined MIC)_/drug A_(MIC alone)_) + (drug B_(combined MIC)_/drug B_(MIC alone)_)

where the FICI ≤ 0.5 was considered synergistic, an FICI between 0.5 and 4.0 was indifferent (noninteracting), and an FICI > 4.0 was antagonistic [26].

#### 2.5.3. Minimum Inhibitory Concentration (MIC) and Minimum Bactericidal Concentration (MBC)

The MIC test was performed on 96-well microtiter plates following the EUCAST guidelines [28]. The microplates were incubated at 90 rpm, and after 18–20 h, one microliter of the well’s contents was streaked on agar. Wells showing 99% inhibition were assigned as the MBC; the concentration below was the MIC [28].

#### 2.5.4. Time–Kill Curve Assay

To investigate the liposomes’ time–kill curve, we monitored their activity for six hours using the CLSI’s guidelines and Brennan-Krohn and Kirby’s methods [29,30]. We used the macro-tube method to investigate the MICs and sub-MICs of the liposomes and free GEN. Ten microliters were collected, diluted (tenfold) in saline, and plated on LB agar at assigned time points. All test tubes and controls were tested in technical triplicates. The results were plotted as the time (X-axis) and Log_10_ CFU/mL of the tested bacteria (Y-axis).

#### 2.5.5. Biofilm Inhibition and Eradication

The inhibition and eradication of biofilm by the Lipo-GEN-THQ formula and the free GEN were tested as proposed by Haney et al. [31], with minor changes. Biofilm reduction and eradication assays were tested in flat 96-well microplates with at least four replicates each. The absorbance (OD_570_) was measured using a spectrophotometer (SpectraMax^®^ M5 Plate Reader, San Jose, CA, USA) after using crystal violet (0.1% *w*/*v*) to quantify the formed biofilm. Lastly, the reduction percentage was averaged and calculated as recommended [22] with the following:

Inhibition percentage (%) = [(OD _(untreated biofilm)_ − OD _(treated biofilm)_)/OD _(untreated biofilm)_] × 100

Next, we tested the liposomes’ ability to eradicate mature biofilm compared to the free GEN [31]. Mature biofilms were grown for 24 h before aspirating the spent media, washing the wells with PBS, and adding Lipo-GEN-THQ formulation or free GEN to their assigned wells. Positive controls (i.e., untreated wells) were replaced with fresh LB broth media. Crystal violet was used to quantify the remaining biofilm, and the absorbance of the de-stained biofilm (with ethanol 70%) was measured (OD_595_) before plotting the above equation’s results.

### 2.6. Lipo-GEN-THQ Cell Adhesion Prevention

#### 2.6.1. Cell Line and Tested Bacteria

We tested the ability of the Lipo-GEN-THQ to prevent the adhesion of *E. coli* (EC-157) to lung tissue using the pulmonary epithelial cell line A549 (ATCC CCL-185™, Manassas, VA, USA). We seeded 2 × 10^5^ cells/well of A549 on a 24-well plate using Advanced DMEM/F-12 (Gibco™, Grand Island, New York, NY, USA) media supplemented with 10% FBS and 1%L-glutamine (without antibiotics) at 37 °C in a humidified 5% CO_2_ incubator until cells reached confluence. In addition, a single colony was inoculated into LB broth and incubated overnight at 37 °C (150 rpm).

#### 2.6.2. Antiadhesion Assay

Following a modified method [32], we tested the Lipo-GEN-THQ formulation and free GEN sub-inhibitory concentrations, which were adjusted using the cell’s media. The bacteria were diluted to 1:100, incubated to the mid-exponential phase, and adjusted to a 10 MOI. Plates were incubated for 1 h to allow for bacterial adhesion. Cells were de-attached using TrypLE Express Enzyme (Gibco™). Samples were collected, diluted, and spotted (10 µL) on agar. After incubation, the bacteria were counted to calculate Log_10_CFU/mL. Two independent experiments were performed, and each plate included each treatment/control in triplicate.

### 2.7. Statistical Analysis

The data were analyzed using GraphPad Prism 10 (macOS Version 10.0.3) and Excel 2013. The means ± standard deviation of the results are expressed, and the statistical differences were determined using a one-way ANOVA followed by Tukey’s (antibiofilm assay) or Dunnett’s (antiadhesion assay) multiple comparison tests.

## 3. Results

### 3.1. Physical Characterization of Lipo-GEN-THQ Formulation: Size, Polydispersity Index, and Z-Potential

**Figure 1:**
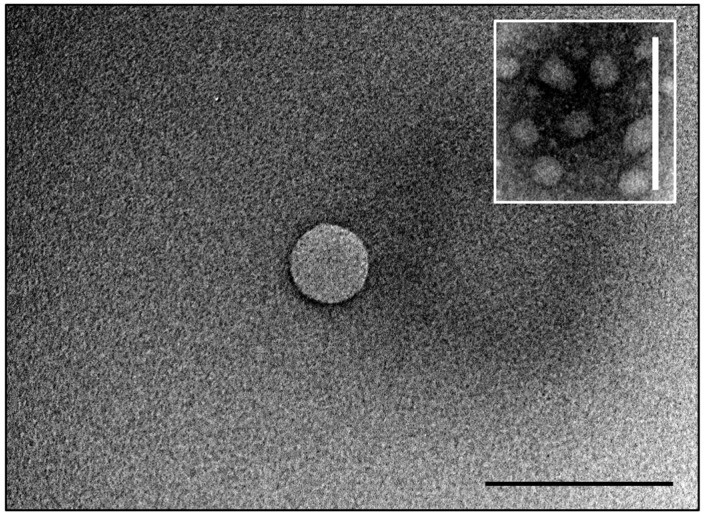
TEM imaging of the spherical Lipo-GEN-THQ formulation at a magnification of 120 K. The inset image (white frame) displays a group of spherical liposomes at a magnification of 80 K; scale bars, 200 nm. Note: the sharpness was enhanced.

**Figure 2:**
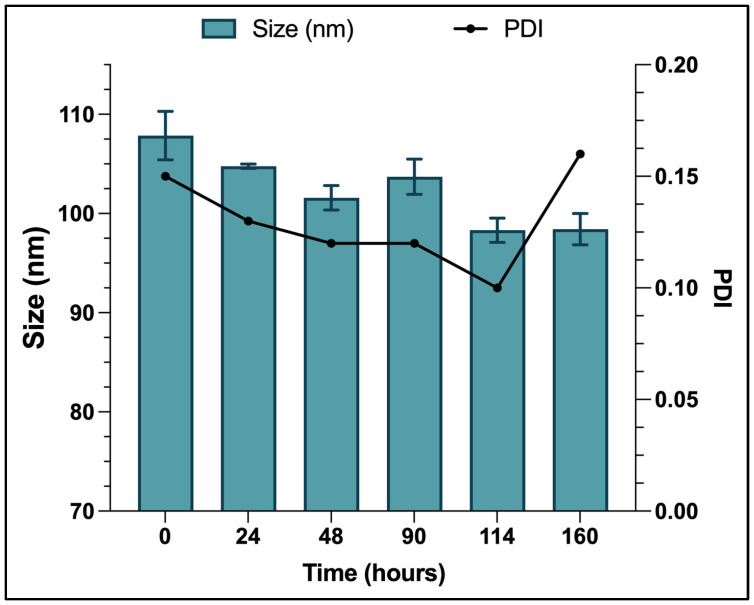
Lipo-GEN-THQ size and PDI stabilities in dH_2_O at room temperature in hours.

The liposomes were characterized using different techniques to confirm their physical characteristics. As shown in Table 1, the loaded liposomes (Lipo-GEN-THQ) had an average hydrodynamic size of 108 nm and a PDI of 0.15. The empty liposomes had a size of 105 nm and a PDI of 0.07. TEM imaging showed evenly distributed particles with clear edges and an average size of 109 nm (Figure 1). The Z-potential measurements showed that the empty and loaded liposomes had negative charges of −44.13 and −16.89 Mv, respectively. Figure 2 shows the liposomes’ size stability in water at room temperature over a week. The liposomes were monodispersed and maintained a PDI between 0.1 and 0.2 throughout the test period.

### 3.2. FTIR Analysis

**Figure 3:**
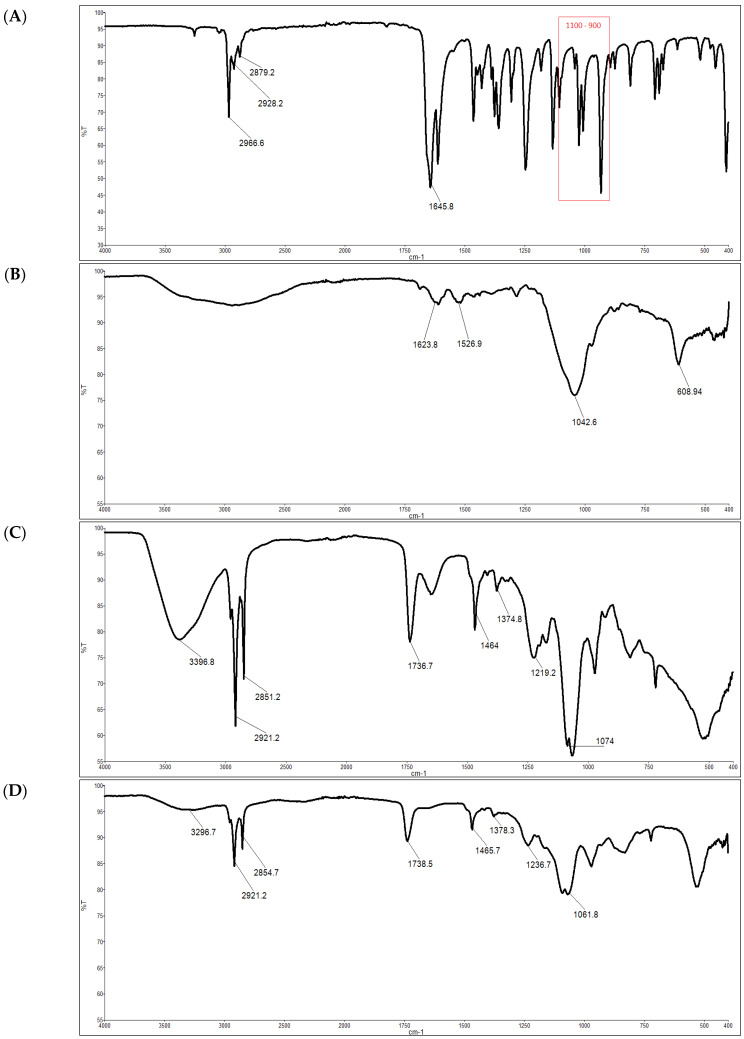
FTIR spectra: (**A**) thymoquinone (THQ); (**B**) gentamicin (GEN); (**C**) empty liposomes; (**D**) liposomal gentamicin–thymoquinone (Lipo-GEN-THQ) formula.

The FTIR spectra of the Lipo-GEN-THQ formulation, empty liposomes, GEN, and pure THQ are displayed in Figure 3. The THQ spectrum, as seen in Figure 3A, reveals major bands at 934 cm^−1^, which is related to the methyl rocking, and 2967, 2926, and 2877 cm^−1^, which correspond to the C-H stretching of the aliphatic groups, attributed to the methyl and isopropyl substituents. The other major band at 1645 cm^−1^ corresponds to the C=O stretching of the carbonyl groups. As shown in Figure 3B, major bands in the GEN spectrum are seen at 1623 and 1527 cm^−1^, which correspond to N-H bending in the amine groups, and at 1042 and 609 cm^−1^, which correspond to the S-O stretch of the sulfur group. Figure 3C shows prominent bands associated with the empty liposomes at 3396 cm^−1^, which correspond to cholesterol’s OH stretching, as well as at 2921 and 2851 cm^−1^, which correspond to the CH_2_ and CH stretching of fatty acid hydrocarbon chains. The C=O stretching of the ester group in the fatty acids produced a characteristic peak at 1737 cm^−1^. The bands at 1219 and 1074 cm^−1^ correspond to the phosphate group’s P-O stretching. The Lipo-GEN-THQ’s spectrum (Figure 3D) had similar peaks as the characteristic bands of the empty liposomes with reduced intensity. The main changes include shifting the phosphate band to 1061 cm^−1^ and broadening the hydroxyl band.

### 3.3. GEN Retention and Lipo-GEN-THQ Formulation Stability

**Figure 4:**
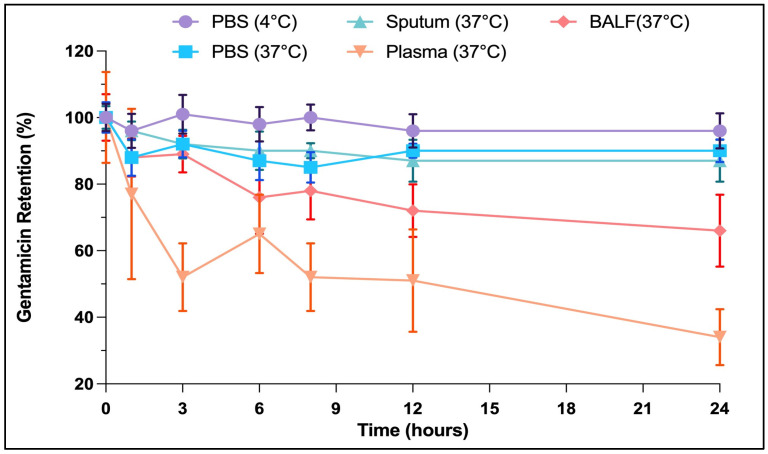
Drug-release assay of Lipo-GEN-THQ under different conditions. The Lipo-GEN-THQ formula was relatively stable in PBS at 4 °C (circle) and 37 °C (square), sputum (upright triangle), plasma (inverted triangle), and BALF (rhombus) and released GEN gradually. The results were averaged and normalized to the mean of the retained GEN at time 0.

The gentamicin was sustainably released from the Lipo-GEN-THQ within 24 h under all conditions to varying degrees (Figure 4). The liposomes were highly stable in PBS at 4 °C and retained 96% of the loaded GEN. It retained 90, 87, and 66% of the GEN at 37 °C after 24 h of incubation in PBS, sputum, and BALF, respectively. The Lipo-GEN-THQ gradually degraded and released approximately 70% of its contents in plasma by 24 h of incubation.

### 3.4. TEM Imaging of Empty Liposome–Bacterial Membrane Fusion

**Figure 5:**
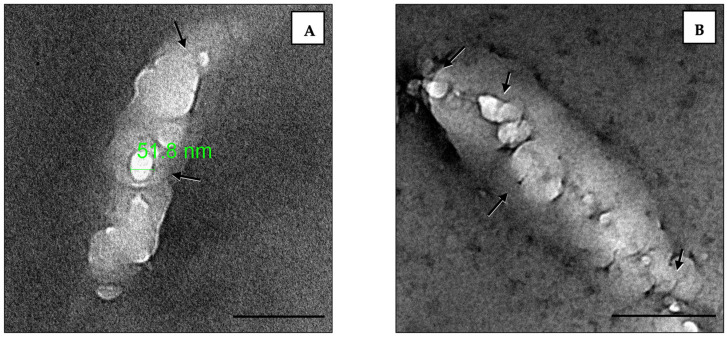
TEM imaging of empty liposomes closely aggregating and fusing to (**A**) *E. coli* ATCC 25922 and (**B**) a susceptible clinical isolate of *E. coli* (EC-157). Arrows are directed toward the liposomes; scale bars, 200 nm. Note: the sharpness was enhanced.

Figure 5 shows how the empty liposomes aggregated and surrounded the bacterial cells, with shrinkage observed. One possible explanation is that empty small nanoliposomes, with their high surface activity and phospholipid structure, have a strong affinity for fusing with the bacterial cell wall. This is one of the advantages of using liposomes as drug scaffolds.

### 3.5. TEM Imaging of Loaded Liposomes (Lipo-GEN-THQ) Effect on Bacterial Ultrastructure

**Figure 6:**
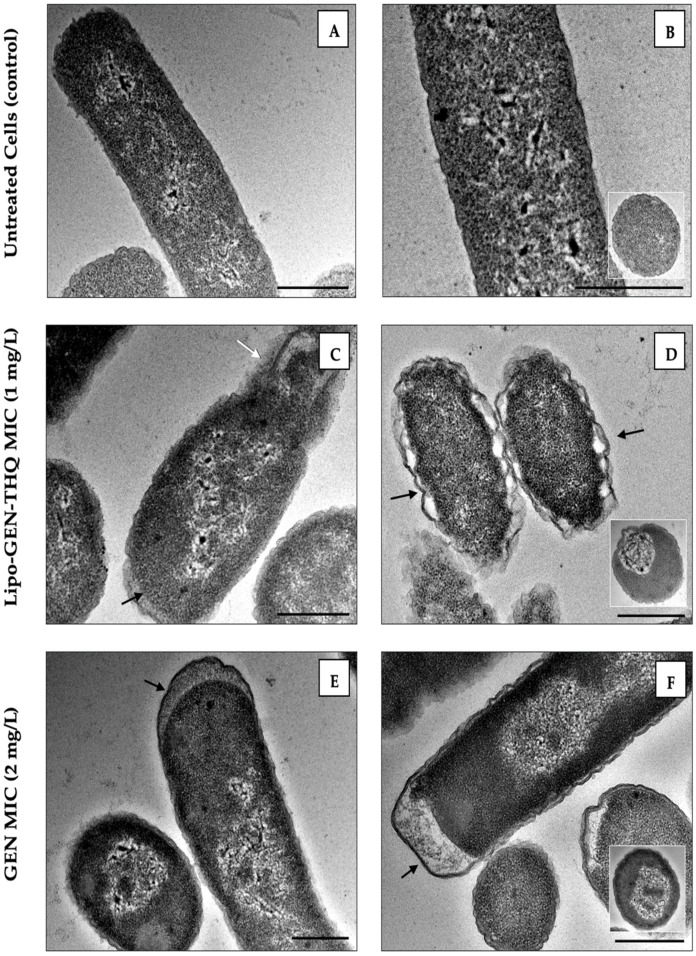
TEM images of a susceptible clinical strain of *E. coil* (EC-157) after one hour of exposure to the MICs of Lipo-GEN-THQ formula and free gentamicin: (**A**,**B**) untreated cells showing intact outer membranes and evenly distributed periplasmic spaces; (**C**–**F**) treated cells, with bacterial cell cross-sections (insets) showing inner changes. Black arrows point to damage to the outer membrane and the extended periplasmic space, and white arrows point to intracellular component release; scale bars, 500 nm. Note: the sharpness was enhanced.

We compared the ultrastructural impacts of the Lipo-GEN-THQ formula’s MIC (1 mg/L) and the free GEN (2 mg/L) on the EC-157 isolate. The untreated cells presented an intact outer membrane and uniform periplasm space (Figure 6). Exposure to Lipo-GEN-THQ and free GEN for one hour deformed the cells, and the cytoplasmic content shrank within the cellular pole (Figure 6C–F). Outer membrane extension and periplasm space separation were observed (Figure 6C–F). The Lipo-GEN-THQ-exposed cells showed intracellular component release (Figure 6C,D). Cross-sections of the treated bacteria reveal an enlarged nucleoid compared to the untreated cells. The Lipo-GEN-THQ produced effects similar to free GEN on the tested *E. coli* clinical strain (EC-157) at half the concentration, indicating higher activity.

### 3.6. GEN Concentration, Checkerboard Assay, Minimum Inhibitory Concentrations (MICs), and Minimum Bactericidal Concentrations (MBCs)

We tested the susceptibility of multiple strains to GEN using the European Committee on Antimicrobial Susceptibility Testing’s standard. The MIC was validated using two ATCC strains. The gentamicin MICs of clinical isolates of *E. coli* and *Klebsiella pneumonia* ranged from 8 to 0.5 mg/L (Table 2). Enterobacterales with MICs > 2 mg/L were considered resistant, and those with MICs < 2 mg/L were susceptible [33].

In the checkerboard assay, the tested concentrations for free THQ and GEN ranged from 1250 to 19.5 mg/L and 64 to 0.06 mg/L, respectively. Combining GEN and THQ reduced GEN’s MIC against the resistant strain EC-219 from 8 to 4 mg/L and THQ’s MIC from >1250 to 39.06 mg/L, suggesting synergism. Some tested bacteria experienced a two-fold reduction in the GEN MIC after combining GEN and THQ (Table 2).

The agar well-diffusion assay showed that the GEN concentration inside the liposomes was 30.28 mg/L, representing a 6% encapsulation efficacy of the initial GEN concentration. The liposomes’ drug-loading capacity was 0.26%. The tested MICs of the Lipo-GEN-THQ ranged from 7.57 to 0.01 mg/L. A two-fold decrease in the MICs was recorded in four of the tested strains, and similar activity by the GEN and liposomes was observed against eight tested strains.

### 3.7. Time–Dose Response of GEN vs Lipo-GEN-THQ Formulation at MIC and Sub-MIC Concentrations

**Figure 7:**
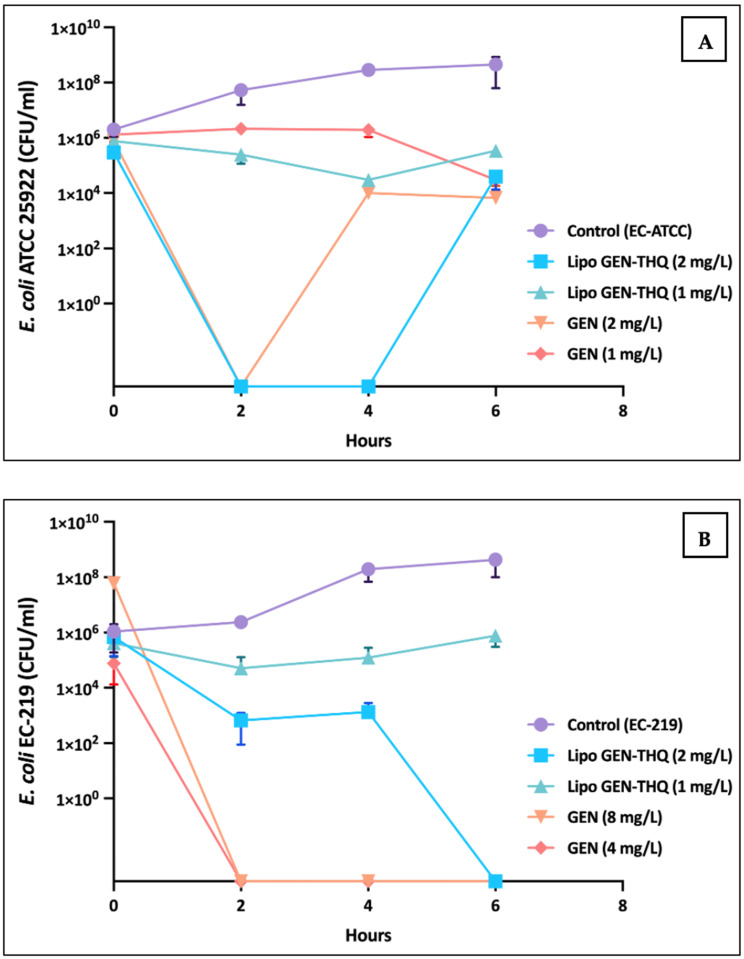
Time–kill curves of different concentrations of Lipo-GEN-THQ and free GEN against (**A**) *E. coli* ATCC 25922 and (**B**) a clinical strain of *E. coli* (EC-219). Untreated cells (control, circle), Lipo-GEN-THQ (MIC, square; Sub-MIC, upright triangle), and free GEN (MIC, upside tringle; Sub-MIC, rhombus).

We tested the Lipo-GEN-THQ and free GEN on two strains of *E. coli* (ATCC and clinical) at different concentrations. Figure 7A shows that both reduced bacterial growth to varying degrees. After 2 h, regrowth occurred with GEN (2 mg/L), while with the Lipo-GEN-THQ (1 mg/L), it occurred after 4 h; both still had a >2 log_10_ reduction compared to the positive control at 6 h. The literature also reported such findings when testing antibacterial agents [34,35]. Figure 7B shows that the liposomes (2 mg/L) eradicated resistant strain EC-219 after 6 h, while higher concentrations of free GEN (8 mg/L) killed bacteria within 2 h.

### 3.8. Biofilm Inhibition and Eradication Assays

**Figure 8:**
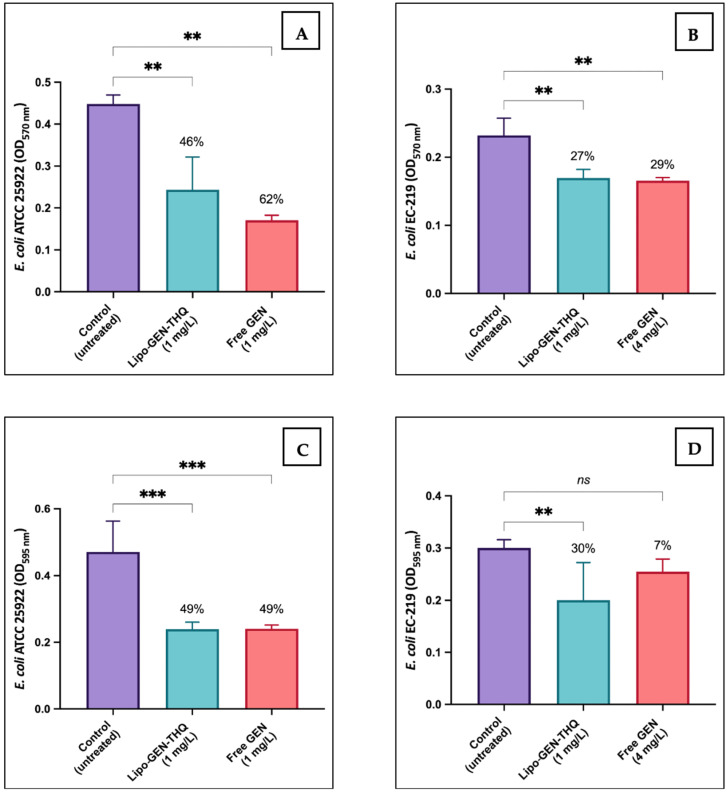
Biofilm inhibition assay of Lipo-GEN-THQ’s and free GEN’s Sub-MICs against (**A**) *E. coli* ATCC 25922 (** *p* ≤ 0.001) and (**B**) *E.coli* EC-219 (resistant clinical strain) (** *p* ≤ 0.002). Biofilm eradication assays of Lipo-GEN-THQ and free GEN against (**C**) *E. coli* ATCC 25922 (*** *p* ≤ 0.0005) and (**D**) *E.coli* EC-219 (resistant clinical strain) (** *p* ≤ 0.01 and *ns* = nonsignificant). The analysis was conducted using a one-way ANOVA test followed by Tukey’s multiple comparison test. The results are the mean of at least quadruplets; bars show the standard deviation.

In Figure 8, we compared the efficacy of the Sub-MICs of Lipo-GEN-THQ formulation and free GEN against bacterial biofilm of *E. coli* ATCC and clinical-resistant strain (EC-219). Both the Sub-MICs of the liposomes and free GEN inhibited 46% and 62% of the biofilm formation in *E. coli* ATCC, but the differences were insignificant (*p* = 0.1892). However, the Sub-MIC of Lipo-GEN-THQ significantly eradicated 30% of the resistant strain’s (EC-219) biofilm (*p* = 0.0081), while the Sub-MIC of GEN at a higher concentration eradicated only 7% of the formed biofilm (*p* = 0.2473).

### 3.9. Antiadhesion Activity of Sub-Inhibitory Concentrations

**Figure 9:**
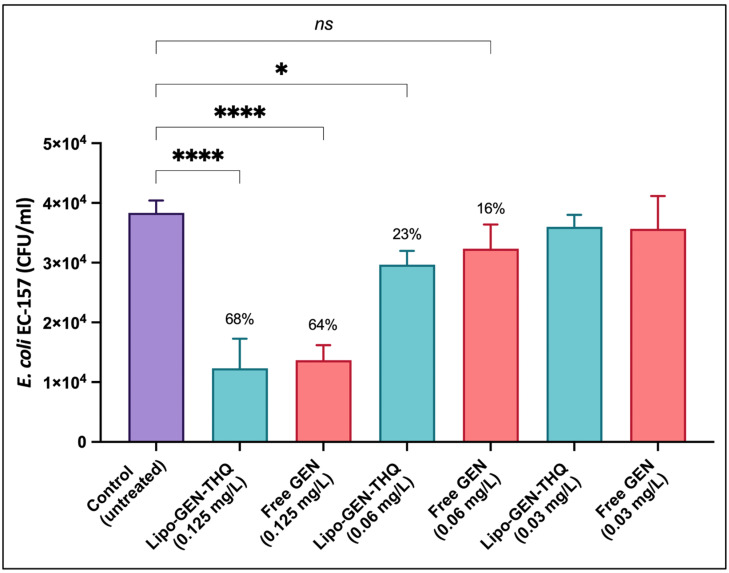
Antiadhesion assays of serial concentrations of the Lipo-GEN-THQ formula and free GEN. Percentages represent the inhibition percentages. The tested concentrations ranged from 0.125 to 0.03 mg/L against the susceptible strain EC-157. **** *p* < 0.0001, * *p* = 0.05, and *ns* = nonsignificant, One-way ANOVA, followed by Dunnett multiple comparison tests.

In this assay, we tested Lipo-GEN-THQ’s and GEN’s ability to prevent EC-157 adhesion to A549 at concentrations ranging from 0.03 to 0.125 mg/L. Figure 9 showed that the Lipo-GEN-THQ (0.125 mg/L) inhibited EC-157 adhesion to A549 similarly (68%) compared to GEN (64%). However, at a lower concentration of 0.06 mg/L, the liposomes significantly reduced bacterial adhesion compared to free GEN (23 vs. 16%).

## 4. Discussion

Antibiotic resistance patterns have resulted in the snowballing of life-threatening infections due to ongoing antibiotic misuse and over-prescription in medicine and agriculture [18,36]. Because of its availability, cost-effectiveness, and broad spectrum, gentamicin (GEN) is widely used in developing low- and middle-income countries, but bacterial resistance and toxicity have limited its usage [37,38,39]. Liposomes are an influential drug delivery system that can reduce cytotoxicity and enhance antibiotic activity [9]. Moreover, loaded antibiotics become available from co-encapsulating other antimicrobials in liposomes, which enhances the antibiotic activity, such as THQ [15]. In this study, we co-encapsulated GEN and THQ in liposomes to increase GEN’s efficacy, which we compared to free GEN using different assays.

Our Lipo-GEN-THQ were spherical in shape, with an average size of 108 nm and a low PDI (0.15), meeting the critical quality attributes (CQAs) for pharmaceutical development established by the US FDA [40]. According to Imran et al. [41], the low PDI of our prepared liposomes indicates a uniform particle size distribution, almost monodisperse in nature. The Microfluidizer system (at 8 MPa) resulted in smaller liposomes, reducing their sizes from >1000 nm to 108 nm, a finding consistent with other studies [41,42]. The zeta potential results show that the empty liposomes had a charge of −44.13 mV, which decreased to −16.89 mV after adding cationic GEN. Similar effects were reported in previous studies [43,44]. Mu et al. [44] attributed it to the electrostatic attraction between the differently charged phospholipids and gentamicin. The negative charge enhances electrostatic stability and reduces aggregation [45], consistent with our DLS and stability results. Other imperative features of the quality of liposomes involve their physical and biological stabilities, drug release rate, and the required time for the drug to reach its target [46].

The physicochemical interactions of the encapsulated GEN and THQ into the Lipo-GEN-THQ formulation were examined by comparing the FTIR spectra of the pure drugs and empty liposomes with the drug-loaded liposomes. The presence of amine and sulfur bands in the GEN spectra was similar to what was reported by Batul et al. [47], and the THQ FTIR spectrum is in agreement with many findings in the literature [48,49]. The major bands related to the phosphate groups and fatty acids were identified in the empty liposome spectra [50]. The remarkable similarity between the spectra of the Lipo-GEN-THQ and the empty liposomes could be due to the high phospholipid ratio relative to GEN and THQ, as previously noted by Ahmad et al. [51]. The slight shift in the phosphate’s characteristic band observed in the spectrum of the Lipo-GEN-THQ compared to the empty liposomes can be attributed to the physical interactions between functional groups from THQ, GEN, and phospholipids, indicating that both drugs were successfully encapsulated within the bilayer structure of the liposomes [47,52]. Mondal et al. [50] found comparable results with the capsaicin–phospholipid complexes compared to free capsaicin. They concluded that the lipids shielded capsaicin, since the FTIR spectra only revealed molecules on the nano-formulation’s surface [50,51].

We tested the size stability of the Lipo-GEN-THQ formula for a week in dH_2_O and for 24 h in different biological media. The Lipo-GEN-THQ were stable under storage conditions and biological media. The Lipo-GEN-THQ were negatively charged liposomes that were freeze-dried and carried an antioxidant (THQ), contributing to their relative electrostatic and chemical stabilities and sustained drug release [45]. The drug-loading capacity was affected by the liposomes’ size and type, lipid composition, drug nature, drug: lipid ratio, and preparation method [53,54]. In our case, the low drug-loading capacity can be attributed to GEN’s highly polarizable nature and high water solubility, which allow for significant amounts of the drug to escape into the aqueous phase during the multiple hydration and rehydration steps required for liposome preparation.

The TEM imaging visualized how the empty liposomes attached to bacteria. The liposomes fused to the bacterial cells closely, similar to previous studies [23,55]. The TEM study of the GEN-THQ-loaded liposomes’ impact on EC-157 caused morphological damage to the EC-157 bacteria even at a lower concentration (1 mg/L) than free GEN (2 mg/L). The damage was also noticeable on the bacterial outer membrane—which Martin and Beveridge observed against *P. aeruginosa—* within 15 min of exposure to 25 mg/L of GEN, and the outer membrane lost 34 and 30% of its proteins and lipopolysaccharides [56]. They noted that GEN created holes in the outer membrane by displacing divalent cations, primarily calcium and magnesium, allowing for greater drug entry into the cytoplasm [56]. Early stages of aminoglycoside activity (in Gram-negative bacteria) start with attaching to the outer membrane’s phospholipids and lipopolysaccharides, then entering the cell to interfere with the protein synthesis process [57]. Cross-sectional images showed a deformed and enlarged bacterial nucleoid compared to the untreated cells, consistent with the exposure to protein synthesis inhibitors [58].

In this study, we tested the antibacterial and antibiofilm activities of the liposomes co-encapsulating gentamicin (GEN) and thymoquinone (THQ) against clinical strains of Gram-negative bacteria. Co-encapsulation with THQ reduced GEN’s MIC two- to fourfold and demonstrated the potential to prevent or eliminate bacterial biofilm at lower concentrations than free GEN. The microbroth checkerboard technique confirmed synergism or indifference against the tested *E. coli* and *K. pneumonia* strains with no antagonism between the free form of GEN and THQ. Previous studies have shown synergism between THQ and GEN against various Gram-positive and Gram-negative bacteria. Dera et al. [59] found similar synergism between THQ and GEN against *K. pneumonia* and *Staphylococcus epidermidis*, while Halwani et al. [60] demonstrated synergism against *S. aureus* but not against Gram-negative bacteria. THQ also exhibits antibacterial and antibiofilm activities in addition to gentamicin’s protein synthesis inhibitory effect. It induces antibacterial action by disrupting multiple cellular processes. This includes interference with lipid synthesis and energy production pathways, damage to the integrity of the bacterial cell wall, prevention of spore germination, generation of reactive oxygen species, and inhibition of protein synthesis [61,62]. In the kill curve assay, regrowth was observed (Figure 7), which may have been caused by the slow release of the drug from the liposome [63], resulting in less available GEN within the early hours of incubation.

Nanotechnology is efficacious in improving antibiotic pharmacodynamics and pharmacokinetics, enhancing antibacterial activity [64,65,66]. Scientists have formulated nano-forms of GEN, such as liposomes [19], lipid–polymer hybrid NPs [67], poly(lactide-co-glycolide) NPs [68], and GEN-coated phosphatidylcholine–chitosan NPs [43]. These nano-GEN particles were effective against bacterial growth and biofilm by co-delivering synergistic drugs, controlling GEN release, and enhancing GEN infiltration into bacterial biofilm [43,67,68]. Moreover, liposomes are excellent for carrying and delivering THQ, since liposomes enhance the solubility and stability of hydrophobic drugs [69].

Bacterial adhesion to the cells is essential to instigate bacterial infections [70]. In our study, concentrations ≥ 0.25 mg/L of Lipo-GEN-THQ formula and GEN had bactericidal effects, prompting us to test concentrations below the Sub-MICs. These results suggest that adding THQ did not affect GEN’s efficacy in Lipo-GEN-THQ. In contrast to our results, a previous study on the antiadhesion effect of the Sub-MIC of GEN against strains of *Pseudomonas* found no significant activity preventing adherence to fibronectin, which is a receptor found on human nasal and bronchial epithelial cells [71]. Research on the antiadhesion activity of liposomal gentamicin is inadequate. Still, less is published on their impact on bacterial adhesions to cells, especially *E. coli*, a common cause of nosocomial pneumonia, mostly in chronically ill patients [72].

## 5. Conclusions

GEN and THQ were successfully co-encapsulated in a liposomal formulation using a simple, reproducible method. The produced liposomes had distinct physical properties, such as small and uniform particle sizes, high surface charges, spherical shape, high integrity, and stability in various biological media. Lipo-GEN-THQ formulation significantly improved antibacterial and antibiofilm activities across a wide range of bacterial strains, including resistant clinical isolates. Lipo-GEN-THQ, on the other hand, retained GEN’s antiadhesion efficacy and inhibited bacterial adhesion to A549 cells in the same way that free GEN did. Lipo-GEN-THQ can achieve therapeutic antibacterial responses at significantly lower doses than free GEN, suggesting a high potential for reducing its known systemic adverse effects. Exploring such positive results through in vivo animal models is strongly encouraged.

## Figures and Tables

**Table 1:** Dynamic and geometric size, PDI, and Z-potential.

Characteristic	Lipo-GEN-THQ Formula	Empty Liposomes
Hydrodynamic size (nm)	107.9 (2.45)	104.5 (1.66)
Polydispersity index (PDI)	0.15 (0.04)	0.07 (0.01)
Geometric size (nm)	109.2 (40.88)	97.0 (35.48)
Zeta potential (Mv)	−16.89 (4.10)	−44.13 (9.30)

The results are recorded as means of at least a technical triplicate (standard deviation).

**Table 2:** Antibacterial activity of the free GEN, Lipo-GEN-THQ formulation, and GEN interactions with THQ (checkerboard assay).

Bacteria	AST **	FICI	Free GEN _(mg/L)_		Lipo-GEN-THQ _(mg/L)_	
			MIC	MBC	MIC	MBC
*Staphylococcus aureus* ATCC 29213	Susceptible	Indifferent	2	4	2	4
*Escherichia coli* ATCC 25922	Susceptible	Indifferent	2	4	2	1
*E. coli* EC-83 *	Susceptible	Indifferent	2	4	1	2
*E. coli* EC-157 *	Susceptible	Indifferent	2	4	1	2
*E. coli* EC-219 *	Resistant	Synergy	8	16	2	4
*E. coli* EC-542 *	Susceptible	Indifferent	2	4	1	2
*E. coli* EC-543 *	Susceptible	Indifferent	2	4	2	4
*Klebsiella pneumonia* KP-19 *	Susceptible	Indifferent	1	2	1	2
*K. pneumonia* KP-47 *	Susceptible	Indifferent	1	2	1	2
*K. pneumonia* KP-86 *	Susceptible	Indifferent	1	2	0.5	1
*K. pneumonia* KP-160 *	Susceptible	Synergy	2	4	2	4
*K. pneumonia* KP-206 *	Susceptible	Indifferent	0.5	1	0.5	1
*K. pneumonia* KP-208 *	Susceptible	Indifferent	1	2	1	2

FICI: fractional inhibitory concentration index; GEN: gentamicin; MIC: minimum inhibitory concentration; MBC: minimum bactericidal concentration. * Clinical strains. ** Antibiotic susceptibility test results are according to The European Committee on Antimicrobial Susceptibility Testing. Breakpoint tables for interpretation of MICs and zone diameters. Version 13.0, 2023. http://www.eucast.org, Access Date: 20 November 2023.

## Data Availability

The original contributions presented in the study are included in the article, further inquiries can be directed to the corresponding authors.
